# A milk-based self-assemble rotavirus VP6–ferritin nanoparticle vaccine elicited protection against the viral infection

**DOI:** 10.1186/s12951-019-0446-6

**Published:** 2019-01-22

**Authors:** Zhipeng Li, Kuiqing Cui, Hong Wang, Fuhang Liu, Kongwei Huang, Zhaojun Duan, Fengchao Wang, Deshun Shi, Qingyou Liu

**Affiliations:** 1 State Key Laboratory for Conservation and Utilization of Subtropical Agro-Bioresources, Guangxi University, Nanning, 530004 Guangxi China; 2 National Institute for Viral Disease Control and Prevention, CDC China, Beijing, 102206 China; 3 National Institute of Biological Sciences (NIBS), Beijing, 102206 China

**Keywords:** Rotavirus, VP6, Vaccine, Ferritin, Nanoparticle, Milk

## Abstract

**Background:**

Rotavirus is the leading cause of severe dehydrating diarrhea in young children and the inner capsid protein VP6 is a potential vaccine candidate that can induce cross-protective immune responses against different Rotavirus strains. The use of ferritin nanoparticles as the scaffold of the antigen can improve the immunogenicity of the subunit vaccines and provide broader protection. We here present a non-live and self-assemble recombinant rotavirus VP6–ferritin (rVP6–ferritin) nanoparticle vaccine.

**Results:**

The rVP6–ferritin nanoparticles were expressed in *E. coli* and self-assembled to uniform spherical structure which similar to ferritin, and oral administration of them induced efficient humoral and mucosal immunogenicity in mice. The nanoparticles were further transgenically expressed in the milk of mice, and pup mice breastfed by transgenic rVP6–ferritin mothers had strongly induced immunogenicity and—compared to pups breastfed by wild type mothers—the proportion of rotavirus challenged pups with diarrhea symptoms, the duration and intensity of the diarrhea, and the deleterious effects on overall growth resulting from the diarrhea were all significantly reduced.

**Conclusions:**

These results suggest that this recombinant VP6–ferritin nanoparticle vaccine can efficiently prevent the death and malnutrition induced by the rotavirus infection in infants and is a promising candidate vaccine for rotavirus.

**Electronic supplementary material:**

The online version of this article (10.1186/s12951-019-0446-6) contains supplementary material, which is available to authorized users.

## Background

Acute gastroenteritis is second only to acute respiratory disease as a cause of severe diarrheal disease and death in infants and young children worldwide and rotavirus is the leading etiologic agent. Vaccination is recognized as the most effective way to prevent the rotavirus infection, and WHO recommends that rotavirus vaccines should be included in all national immunization programs, particularly in countries with high rotavirus gastroenteritis (RVGE) associated fatality rates [1]. Since 2006, two attenuated rotavirus vaccines (RotaTeq and Rotarix) have been licensed in > 100 countries worldwide [2, 3], and helped to protect millions of infants from rotavirus infection. These live-attenuated vaccines have a strong protective effect, but they are known to lead to side-effect like vaccine-derived infection, intussusceptions, and low fever, and a small number of patients may face the risk of spontaneous reversion to virulence [4–6]. Non-live vaccines including subunit vaccines are now understood as powerful alternatives and the highly conserved rotavirus inner capsid protein 6 (VP6) has been discussed as an efficient candidate vaccine [7–9].

As the middle layer protein of rotavirus, VP6 is the main component of viral structure protein, and content for 51% of the total viral protein [10]. Although the protective mechanisms of VP6 have not been completely clarified, it can induce heterotypic cross-protective rotavirus immunity responses and confer protection against rotavirus in animal models [11–13]. Subunit vaccines reduce the risk of side effects such as spontaneous reversions of attenuated vaccines and denaturing of antigenic peptides with inactivated vaccines [14], while the problem is that subunit vaccines in general are not as immunostimulatory as the whole organism vaccines [15], and usually require cholera toxin (CT), CpG or heat-labile enterotoxin (LT) as the adjuvants. It is exciting that the use of nanotechnology made Nano-vaccines to be effective antigen delivery systems [5] and the highly symmetric and self-assembling ferritin nanocage has presented an attractive target for vaccine development. There has been successful application which fused Nano-cages formed by the *Helicobacter pylori* ferritin protein with peptides derived from HIV-1 proteins (the Tat peptide) and influenza virus proteins (haemagglutinin) to develop vaccines that effectively elicit potent humoral responses and induce the production of broadly neutralizing antibodies against these pathogens [16, 17].

As rotavirus mainly infects the infants, suitable dosage forms and methods of inoculation should be considered. Breast milk provides an ideal source of nutrition for infants and promotes rapid growth and the development of gut function [18, 19]. Bovine milk has been used as the main supplementary food for infants and attempts have been made to add human proteins, such as α-lactalbumin [20], lactoferrin [21, 22], and lysozyme [23] in milk by producing genetically modifying dairy cows to promote the nutritional content. While the most important difference between milk and breast milk lies in that the rich immunoglobulins in breast milk provide immunological protection to the infants and young children against multiple pathogenic microorganisms [24, 25]. It is worth noting that the immunoglobulin cannot be added directly into milk as the serum-derived antibodies often fail to prevent pathogens from infecting the intestinal mucosa. One solution may be that we can express the antigenic proteins, such as subunit vaccines, in the milk to induce the body to produce autoimmunity and secretes antibodies to prevent the infection. It is now well-established that mammary glands can be used to transgenically produce pharmaceutical proteins [26]. Advantages of using transgenic animals to produce recombinant proteins include large production yields at a relatively low cost, as well as high quality—recombinant proteins produced in animals often undergo complex post-translational processing to the native biological activity [26, 27]. Milk-based pharmaceutical production is already underway: the FDA-approved and commercially available clotting disorder biologic drug ATryn is derived from the milk of goats, and the hereditary angioedema drug Ruconest is produced in the milk of transgenic rabbits [28]. Thus, provided that a recombinant vaccine did not require extensive post-production purification or chemical processing steps prior to administration, milk-based production appears as an extremely attractive technological solution for producing enteral vaccines that could be directly administered in their very production medium (milk) to help protect infants against infections.

In this study, *Helicobacter pylori* ferritin (Hpf) nanoparticles was designed as the antigen delivery system, and rotavirus VP6 protein was present on the surface of the nanoparticles. Seeking to design a rotavirus vaccine that would be suitable for both mammary-gland-based production and milk-based administration, we present an experimental vaccine in the milk and the immunogenicity and immune protection of this vaccine was evaluated in mice model.

## Results

### Synthesis of a codon-optimized rVP6 and rFerritin gene

A number of codons were altered to match the codon usage preferences of mammals and the nucleotide coding sequence was modified to yield a new sequence called rVP6 or rFerritin. The rVP6 and rFerritin gene was then linked by a space sequence ‘AGCGGCGGC’ and named rVP6–ferritin (Additional file 1: Figure S1). The nucleotide sequence of the rVP6 gene was 83.75% homologous to the original VP6 gene. The amino acid sequence encoded by the modified rVP6 and rFerritin was identical to that of the original gene to ensure retention of the antigenicity and structural features of the transgenically expressed protein.

### Prokaryotic expression, purification and TEM analysis

**Fig. 1:**
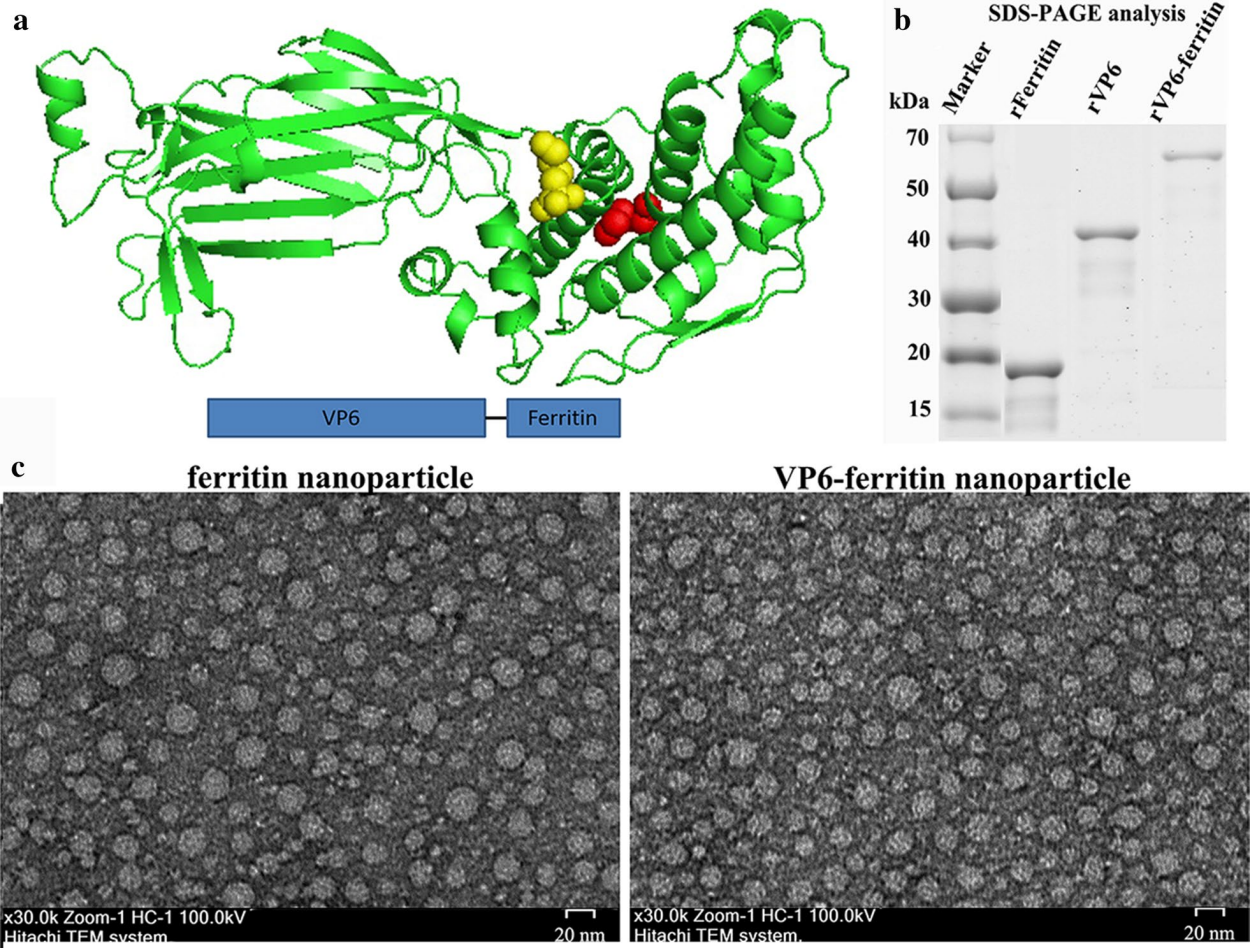
Prokaryotic expression, purification and transmission electron micrographic images of recombinant protein. **a** A subunit of VP6–ferritin (PDB); detection of recombinant protein by Western blotting with anti-VP6 antibodies. **b** SDS-PAGE analysis of the purified and digested recombinant protein. **c** TEM images of negatively stained rFerritin (left) and rVP6–ferritin (right)

The *rFerritin*, *rVP6* and *rVP6*–*ferritin* genes were inserted into pET-32a vector between the *Nco*I and *Xho*I sites, and the rVP6 was designed to fused on the N-terminus of ferritin (Fig. 1a). Western blot analysis with anti-his-tag antibody showed that the molecular weight was about 40 kDa, 60 kDa and 80 kDa respectively (Additional file 1: Figure S2). They were then purified by Ni-affinity FPLC and the his-tag was removed by enterokinase digestion (Fig. 1b). Further analysis showed that the rVP6–ferritin protein self-assembled to spherical particles (~ 20 nm) as visualized by transmission electron microscopy (TEM), which is similar to the ferritin (Fig. 1c).

### Molecular weight and hydrodynamic size analysis of the rVP6–ferritin subunit

The molecular weight of rVP6–ferritin subunit was obtained by MALDI-TOF/MS. Results showed that the molecular weight of the subunit is 67,098 ± 1.0 Da, which is in good agreement with the theoretical value of single subunit (Additional file 1: Figure S3A). The difference of the molecular weight between the rVP6–ferritin and ferritin subunit (theoretical value: 21,421 Da) indicates that VP6 protein was combined with the ferritin subunit.

The hydrodynamic size and zeta potential was analyzed by dynamic light scattering (DLS). The size distribution of the rVP6–ferritin nanoparticles was consisted by two mainly sizes, among which 78.9% are 83.00 nm and the remaining 21.1% are 19.61 nm (Additional file 1: Figure S3B). Though part of the nanoparticles have similar diameter with that of TEM, most of the nanoparticles showed larger size. This can be due to that DLS gives a hydrodynamic size that corresponds to the core and the swollen corona of the micelles, whereas TEM often gives a size of the core for micelles in a dried state as the corona with low electronic density is not visible. Thus, though the branches of VP6 subunit on the surface of ferritin nanoparticle were invisible by TEM, it may affect the hydrodynamic size in DLS analysis. Another reason may be that the nanoparticles were assembled together in the solvent. The zeta potential was 2.79 mV.

### Antibody responses in mice after immunized by the recombinant protein

**Fig. 2:**
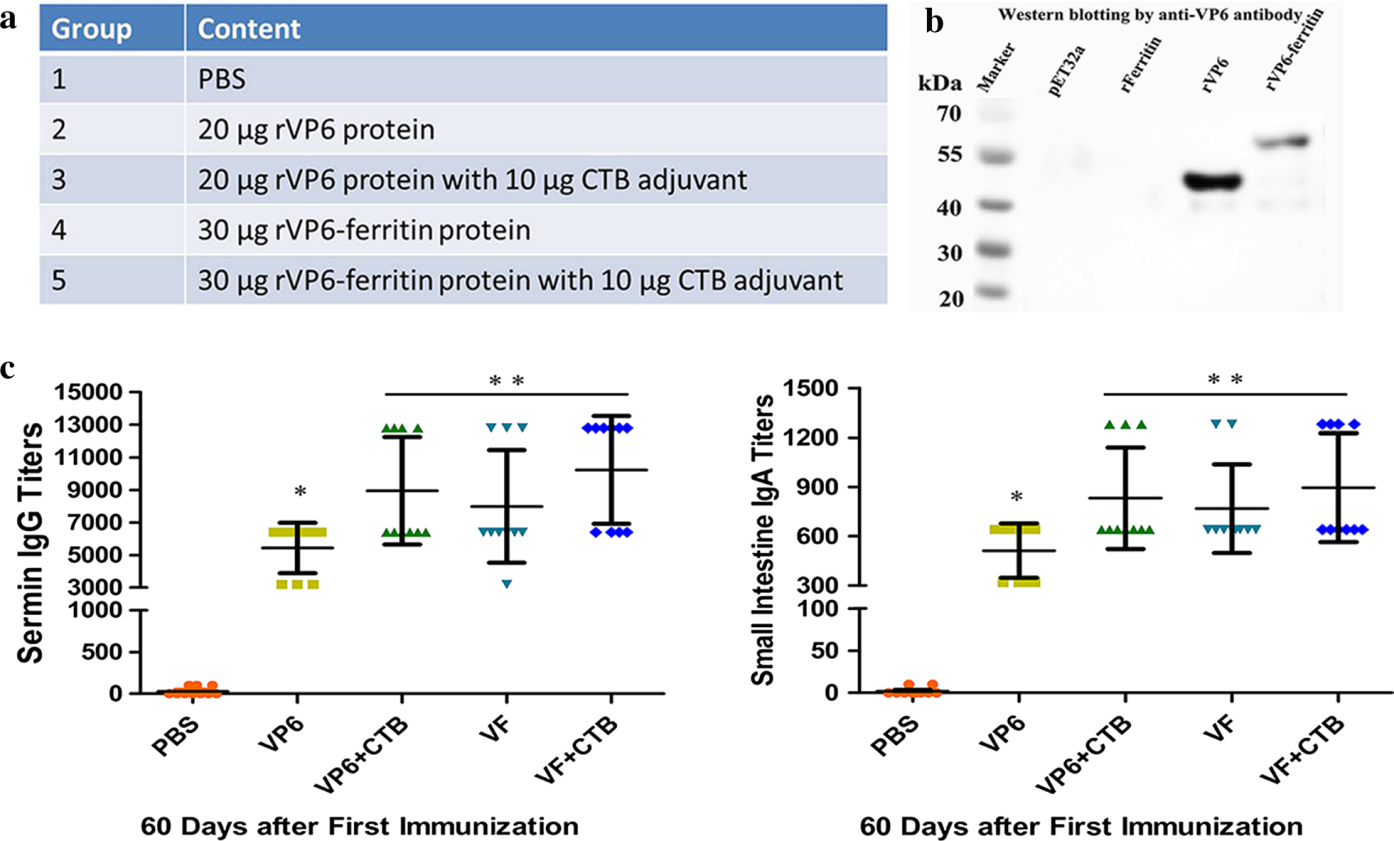
Antibody responses in mice orally immunized by recombinant protein. **a** Oral administration controls of mice. **b** Detection of recombined protein by Western blot with anti-VP6 antibodies. **c** Anti-VP6 IgG (left) and IgA (right) antibody titers determined by ELISA. VF = VP6–ferritin. The results are presented as mean ± SEM. Values marked with * are significantly different (P < 0.05)

To detect whether the recombinant protein is capable of inducing a high level immune response, mice were orally administrated with the recombinant protein with or without the CTB adjuvant, along with five control groups (Fig. 2a). Detection of recombinant protein by western blotting with anti-VP6 antibodies showed that the rVP6 and rVP6–ferritin protein can bind the anti-rotavirus VP6 antibodies specifically, while no bound was detected in the sample of pET-32a control and ferritin (Fig. 2b). These results indicate that the rVP6 and rVP6–ferritin protein has similar antigenicity to rotavirus VP6, while the his-tag and rFerritin won’t induce VP6 specific immunization. Mice were gavaged with purified proteins once a week for 4 weeks and sacrificed to obtain the serum and small intestines samples to analyze anti-VP6 IgG or IgA titers on day 60 after the first immunization. Results showed that, oral administration of rVP6–ferritin in mice induced antibody titers higher than those of rVP6 peptides (8000 ± 1093 vs. 5440 ± 488.8 anti-VP6 IgG titers and 1152 ± 248.8 vs. 608 ± 88.6 anti-VP6 IgA titers) with statistical significance (p = 0.0251 for IgG and p = 0.0051 for IgA antibody, N = 10). Though mice gavaged rVP6 or rVP6–ferritin together with CTB adjuvant demonstrate higher anti-VP6 IgG or IgA titers than those of rVP6–ferritin, no statistical significant difference was found between them. The group immunized with PBS did not produce any anti-VP6 antibodies (Fig. 2c). Results indicate that the use of ferritin enhanced the immune response of rVP6. Subsequently, rVP6–ferritin protein was used as the antigen protein as its efficient antigenicity.

### Acute toxicity analysis of the recombinant proteins and nanoparticles

As the nanoparticles were macromolecule proteins, we don’t think this nanoparticle can be absorbed into the circulatory system and induce acute toxicity in the blood. Therefore, the particles were mainly in the intestine and the acute toxicity effects of VP6–ferritin nanoparticles were evaluated by haematoxylin–eosin staining. Results showed that the intestinal mucosa of mice gavaged with the nanoparticles were as healthy as the PBS control group (Additional file 1: Figure S4), which indicated that the nanoparticles were harmless to the intestinal of mice. The mucosa and microvilli of the small intestine are as normal as the control group. Moreover, all of the mice gavaged with the rVP6–ferritin nanoparticles and pups breastfed by milk containing rVP6–ferritin were as healthy as the mice did not administrated with the nanoparticles in their following life. There was no significant difference about the growth and development (e.g. body weight, reproductive capacity) between the mice oral administrated with the nanoparticles or not (Data not shown for these results were obtained from the daily feeding of the mice without systematic testing).

### Production of the rVP6–ferritin nanoparticle vaccine in the milk of transgenic mice

**Fig. 3:**
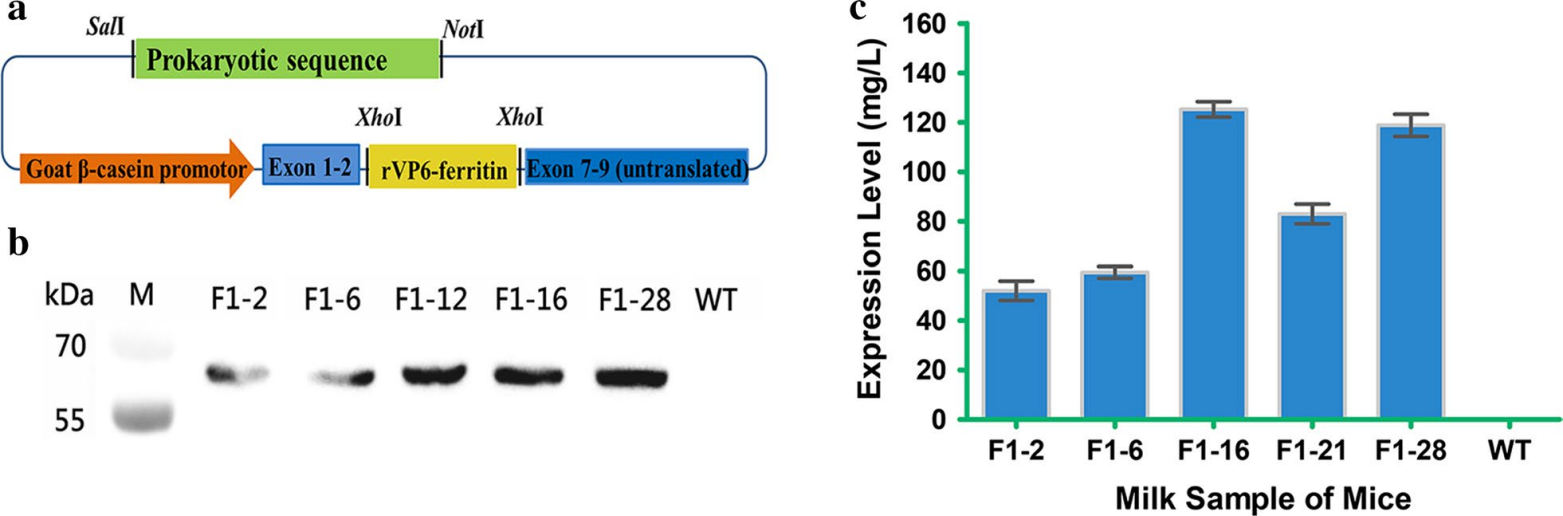
Milk specific expression of rVP6–ferritin fusion protein. **a** Schematic diagram of mammary gland specific expression vector. **b** Identification of rVP6–ferritin proteins expressed in the milk of transgenic mice by Western blot. **c** Quantification of rVP6–ferritin proteins expressed in the milk of transgenic mice by ELISA. The results are presented as mean ± SEM

The *rVP6*–*ferritin* gene was inserted into the *Xho*I site of gland expression vector pBC_1_, and the prokaryotic sequences can be removed by digesting with *Not*I and *Sal*I (Fig. 3a). Transgenic mice were genotyped via PCR analysis of tail biopsy DNA samples, and five transgenic founder mice were obtained containing three female and two males (Additional file 1: Figure S5A). Southern blotting was used for further confirmation of transgenic genotypes and showed the expected 1.7 kb DNA fragment present in the genome of each of the founder mice (Additional file 1: Figure S5B). Moreover, RT-PCR confirmed the mammary-gland-specific expression of the *rVP6*–*ferritin* construct (Additional file 1: Figure S5C). Western blotting further confirmed the accumulation of the rVP6–ferritin fusion protein in milk, and the expression of the rVP6 protein in the transgenic mice was quantified by comparison with the standard curve (Additional file 1: Figure S6). Results showed that specific hybridization bands with molecular mass about 67 kDa were detected by anti-VP6 antibody (Fig. 3b), and the quantification is in the range of 52.04 to 125.25 mg/L (Fig. 3c).

### Antibody responses in mice after oral administration of milk from rVP6–ferritin transgenic mice

**Fig. 4:**
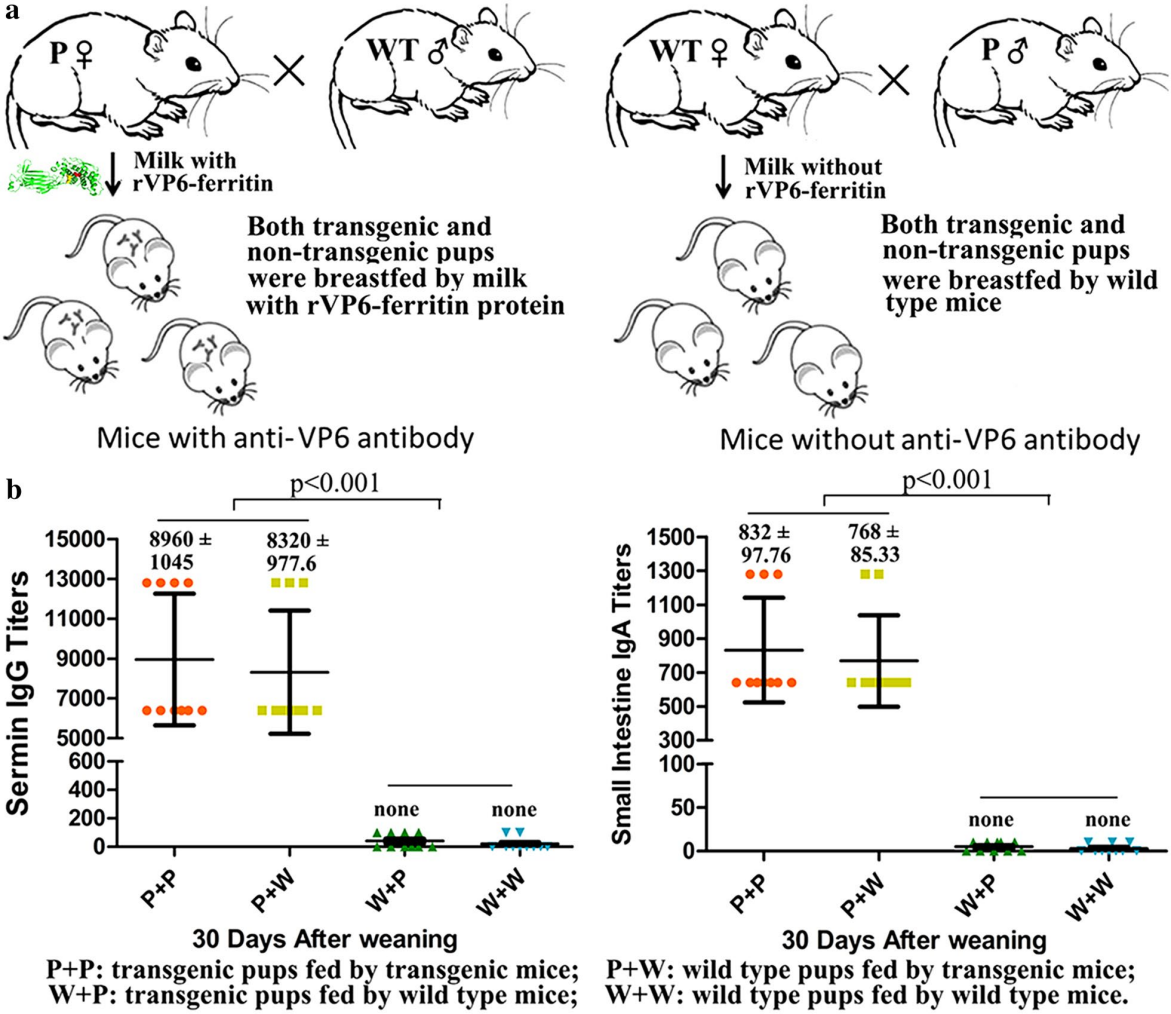
Immunogenicity analysis of rVP6–ferritin fusion protein. **a** Schematic diagram of mice breeding program. **b** Serum IgG (left) and mucosal IgA (right) antibody titers determined by ELISA in mice breastfed by milk from transgenic mice and wild type mice. P indicate transgenic positive mice; W indicate wild type mice. The results are presented as mean ± SEM

The heterozygous mice were mated with wild type mice to obtain both transgenic positive and negative pups, so that both transgenic positive and negative pups can be breastfed by transgenic mother and wild type mother without artificial participation (Fig. 4a). The pups were allowed to breastfeed for 3 weeks and ELISA was used to quantify the titers of serum IgG and mucosal IgA antibodies. Whereas both transgenic and wild type pups that breastfed by transgenic mice produced specific anti-VP6 IgG and IgA antibodies (8320 ± 977.6 anti-VP6 IgG antibodies and 768 ± 85.33 anti-VP6 IgA antibodies), none of the pups breastfed by wild type mice generated any serum IgG and mucosal IgA antibodies (Fig. 4b). These experiments established that rVP6-specific immunogenic responses can be successfully induced in mice breastfed by milk containing rVP6–ferritin.

### Rotavirus challenge experiments

**Fig. 5:**
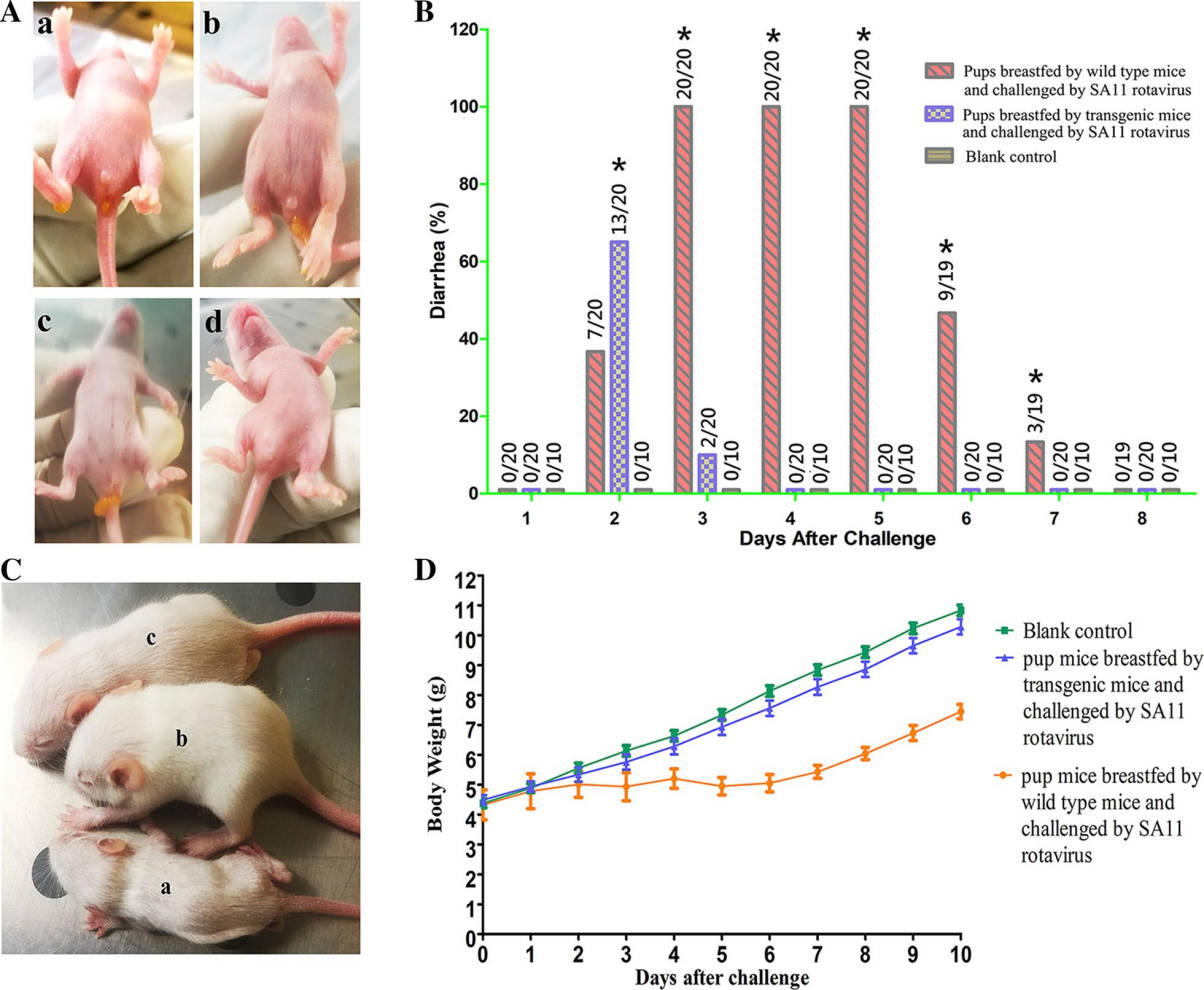
Protection against rotavirus challenge in milk immunized pup mice. **A** Photos of pups on the 3rd day after challenge; a: wild type pups fed by wild type mother mice; b and c: wild type and transgenic pups fed by transgenic mother mice; d: blank control. **B** Diarrhea rate of pup mice after rotavirus challenge. **C** Photos of pups on the 10th day after challenge. a: pups fed by wild type mother; b: blank control; c: pups fed by transgenic mother. **D** Change in body weight after virus challenge. Each data point represents the mean change in body weight from day 0 (pre-challenge) with SEM (n = 10)

The anuses of diarrheic mice were reddish and adhered with feces, and the diarrhea symptomatic between normal and diarrheal mice was obvious. Pups without challenge did not show any diarrhea symptoms at any point of these experiments. Rotavirus-challenged pups breastfed by wild type mothers suffered watery diarrhea for many days after rotavirus exposure (Fig. 5A). In sharp contrast, the rotavirus-challenged pups breastfed by rVP6–ferritin transgenic mice had only mild cases of diarrhea that did not typically last more than about 2 days. Specifically, no diarrhea symptoms were observed in any of the groups on the first day after inoculation of virus. Among the pups fed by rVP6–ferritin transgenic mice, 13/20 suffered mild diarrhea on the second day, with 11 of these recovering by the afternoon of the third day (the 2 remaining were recovered by the fourth day). Among the pups fed by wild type mothers, however, 7/20 suffered severe diarrhea on the second day, and 20/20 of them suffered diarrhea until the fifth day; half of these had recovered by the sixth day, but one of the pups died of malnutrition (Fig. 5B).

Apart from their diarrhea, we also noticed significant differences in the body weights of the pups of the different experimental groups: rotavirus-challenged pups fed by wild type mothers were skinny and malnourished, whereas rotavirus-challenged pups fed by transgenic rVP6–ferritin mothers appeared as healthy as the control pups that were naive to rotavirus challenge (Fig. 5C). Analysis of the change of body weight showed that rotavirus-challenged pups fed by wild type mice suffered from significant growth retardation, while the pups fed by transgenic mice did not (Fig. 5D). In brief, compared to pups fed on milk lacking rVP6–ferritin, the proportion of pups with diarrhea symptoms, the duration and intensity of diarrhea, and the deleterious effects on overall growth resulting from the diarrhea were all significantly reduced in the pups fed with milk containing rVP6–ferritin.

## Discussion

Here we report a ferritin nanoparticle-based rotavirus vaccine produced in mammal gland, and breast feeding of this milk induced specific humoral and mucosal immunity to protect the pup mice against rotavirus infection and relieve the symptoms of diarrhea. Although there is no doubt that the two available, live-attenuated vaccines are efficacious in preventing severe rotavirus disease, improved rotavirus vaccines and the development of better mucosal adjuvants or delivery systems is needed to overcome the current rotavirus vaccine deficiencies, particularly their inability to produce serotype-specific protection and the risk of occurring reverse mutation to a virulent virus. Non-live vaccines should instead the live-attenuated vaccines, and VP6 has been discussed as an alternative vaccine candidate [7–9]. Though the correlates of protection against rotavirus infection are not fully understood, the high level of serum rotavirus IgA antibody targeted to the rotavirus inner capsid protein VP6 has been considered as the best surrogate marker [29, 30]. The IgA antibodies direct to VP6 can inhibit rotavirus replication intracellularly [31] and prevent or resolve rotavirus infection in mouse model, and the polymeric IgA can move the rotavirus into the gut lumen via transcytosis from the basolateral membrane to the apical membrane of enterocytes [32].

Though safety and efficiency, the subunit vaccine is generally required adjuvants for improving the immunization and the ferritin nanoparticle provide another strategy for rational design of immunogens. Ferritin nanocages can enhance the immunogenicity of antigens by displaying multiple antigens on their outer surface in an orderly manner, which similar to the whole organism vaccines. This hypothesize was based on the premise that highly ordered repetitive arrays induce yet stronger immune responses [16, 33]. There has been successful application that fused Tat peptide and haemagglutinin on the amino terminus of ferritin, which with proper assembling and elicits a potent humoral response [16, 17]. Moreover, the ferritin-based nanoparticle vaccine improved the potency and breadth of influenzas virus immunity. The rVP6–ferritin nanoparticle in our research also self-assembled to homogeneous spherical particles and elicited higher anti-VP6 specific antibody titers. It seems that the inserted rVP6 peptide was multiple conformations on the nanocage surface, which similar to the inserting of *N. gonorrhoeae* peptides [34], with no visible viral spikes on the nanocage surface by the TEM analyze. The resolution of the photos by TEM should be improved for more detailed observation and further crystal structure analysis may help to understand the mechanism of the functional nanoparticles. However, the structure of VP6 protein is not absolutely strict to achieve efficient vaccination [35] and the improved immunogenicity of the rVP6–ferritin nanoparticle supports this standpoint.

Edible vaccines are potential developing direction of vaccination, and much effort has been made on the rotavirus vaccines. Production of rotavirus VP6 in transgenic plants such as potato, alfalfa, tabaco and maize has been achieved, and gavage of the extracts with adjuvants (CT, LT or CpG et al.) in lactating mice induced passive protection of offspring [36–39]. These are successful effort for developing edible vaccines, but the extracts and adjuvant are not welcomed by the infants and the purification is expected to be complex and difficult. While as the main nutritional food for infants, milk is a perfect medium for enteral vaccine which does not need any complex process or purification. In a previous study, the rotavirus recombinant VP2 and VP6 proteins were co-expressed in the milk of transgenic rabbit, and gavage of this milk with CT (cholera toxin) adjuvant induced a significant reduction in viral antigen shedding in adult mouse model [35]. Though this is a successful attempt to produce milk-based vaccines, it still needs CT adjuvant as the adjuvant as the subunit vaccines are not as immunostimulatory. In this report, pups breastfeeding by the milk containing rVP6–ferritin were induced efficiency humoral and mucosal immunity, which prevent the rotavirus infection and relieve the symptoms of diarrhea, without the using of any adjuvant. The ferritin-based vaccine produced in milk of mice provides an ideal edible vaccine model. Moreover, the ferritin nanocages can be reversibly disassembled when the pH becomes extremely acidic and self-assemble in a shape memory fashion when the pH returns [40], which made the rVP6–ferritin as nanocages in the intestine. It is exciting that the ferritin nanocages are resistant to denaturants, including heating to high temperatures [41], which is necessary for transgenic milk to be processed into milk powder or ready to drink, as much higher temperatures must be tolerated during the spray-drying process or Pasteurize.

Although not a mature rotavirus vaccine, this approach provides a novel strategy for vaccine design and production. Moreover, the synthetic nanoparticles are fully recombinant, eliminating the need to produce potentially dangerous virus in eggs or in cell culture, and allowing for modifications that improve immunogenicity which would otherwise not be tolerated in replication-competent viruses currently used to manufacture vaccines. This milk-based self-assemble nanoparticle vaccine therefore represents a foundation for a new generation of rotavirus vaccines and could be adapted to create analogous oral vaccines for other pathogens, especially those pathogens that infect the gastrointestinal. In addition, as an efficient and inexpensive recombinant protein expression system, this approach could also be applied to development parenteral vaccines for a wide variety of pathogens by purifying the nano-vaccine from the milk.

## Conclusions

Our study successfully establishes that the rVP6–ferritin can self-assembled to spherical nanocage and induce significant higher immune response than rVP6 peptide. Moreover, edible vaccines can be produced in mammary glands and that both immunogenicity and protective effects against rotavirus can result from feeding on the vaccine-containing milk. Looking forward, mice were used as animal model for investigating the feasibility of this nanoparticle vaccine, and we are making efforts to build on the milk-based vaccines in milk of larger animals like goats and cows. Indeed, the ability to produce functional vaccines directly in the milk of domesticated animals could even define a new public health delivery regime.

## Methods

### Design of the rVP6–ferritin fusion construct and expression in *E. coli*

The gene encoding the VP6 protein of human rotavirus group A (Gene Bank accession No: AB022768.1) and the *Helicobacter pylori* non-haem iron containing ferritin (Gene Bank accession No: NP223316) was synthesized (mammalian-preferred codon usage and ligated into the pUC19 vector by Shanghai Sangon Company). The sequence encoding the signal peptide of goat β-casein (M-K-V-L-I-L-A-C-L-V-A-L-A-I-A) was fused in front of the VP6 protein sequence to generate a secreted protein; a serine-glycine-glycine (S-G-G) spacer was fused between the rVP6 and the rFerritin sequence.

To evaluate the self-assembly and the antigenicity of the rVP6–ferritin protein, the *rFerritin*, *rVP6*, and *rVP6*–*ferritin* genes were inserted into the pET-32a vector between the *Nco*I and *Xho*I sites, and the plasmids were transformed into *E. coli* BL21 (DE3) cells. The expressed rFerritin, rVP6, and rVP6–ferritin proteins were purified by His-tag affinity chromatography, enterokinase, and fast protein liquid chromatography (FPLC). Tris-buffer containing 30% glycerin (v/v) was used to store the particles, and the protein was buffer exchanged into PBS at home temperature before the immunization experiment. Samples of the purified rFerritin, rVP6, and rVP6–ferritin proteins were incubated at 100 °C for 10 min in loading buffer and analyzed by SDS-PAGE using a TGX Stain-Free FastCast Acrylamide Kit 10% (Bio-Rad, 161-0183). The separated proteins were transferred onto nitrocellulose membranes for western blotting using an Electro-transfer unit (Bio-Rad). After blocking with phosphate-buffered saline (PBS) containing 0.5% Tween- 20 (PBST) and 5% skim milk for 2 h at room-temperature, the membranes were reacted with mouse anti-rotavirus VP6 antibody (Abcam, ab181695, 1:500) at 4 °C overnight, followed by HRP-labeled rabbit anti-mouse IgG (1:5000) (Proteintech Group, Inc). After washing, the reaction was developed using the enhanced chemiluminescence detection system (Pierce ECL, Thermo).

### Molecular weight of rVP6–ferritin subunit by mass spectroscopy

The exact molecular weight of protein subunit was obtained by MALDI-TOF/MS (ultraflextreme TOF/TOF) in refractive positive-ion mode. The sample was prepared in water/trifluoroacetic acid (100:0.1 v/v). Data were collected between 20,000 and 90,000 Da.

### Dynamic light scattering (DLS) analysis of rVP6–ferritin nanoparticles

Dynamic light scattering experiments were performed on a Malvern Zetasizer Nano ZS in disposable polystyrene micro cuvettes (VWR) using 10 mL of freshly prepared sample solution (0.5 mg/mL, pH = 7.4). After equilibration to 25 °C, three measurements were performed with the instrument optimizing the number of runs for each measurement. The refractive index (RI) of the dispersant (preset: water) was set to 1.330 and the viscosity (cP) to 0.8872, respectively. The RI of the particle was set to 1.45. The absorption of the protein was set to 0.001, both attenuator and measurement position were controlled by the instrument.

### Electron microscopic analysis

Purified rFerritin nanoparticles and rVP6–ferritin nanoparticles were negatively stained with phosphotungstic acid and ammonium molybdate, respectively, and images were recorded on a Tecnai T12 microscope (FEI) at 100 kV with a CCD camera (AMT Corp.).

### Oral immunization of mice and immunogenicity assay

Fifty female BALB/c mice (6 weeks old; Experimental Animal Center, Guangxi Medical University, Nanning, China) were divided into five groups and immunized orally at weekly intervals for 4 weeks with the recombinant proteins. Bacterial cholera toxin B subunit (CTB, C9903, Sigma) is one of the adjuvants approved for human use in vaccines and has been used as the adjuvant for rotavirus VP6 vaccine in a previous study [39]. CTB therefore was used as the adjuvant in two of the groups in this study. The mice were fed 100 µL phosphate buffer (0.05 M PBS) containing 20 µg rVP6 protein (20 µg of VP6 antigen) or 30 µg rVP6–ferritin proteins (20 µg of VP6 antigen) with or without 10 µg CTB. These concentrations were experience dose based on several previous studies [36, 39]. One group of mice was fed with PBS only as the negative control.

On day 30 after the final immunization, all of the mice were sacrificed, and sera samples were collected for the measurement of anti-VP6 IgG titers; the small intestines were removed and the lumen of the intestine was washed with 1 ml PBS buffer (pH 8.5, containing 10 mM PMSF) to collect any mucosal antibodies for analyzing anti-VP6 IgA titers. The levels of anti-VP6 IgG and IgA were assayed by ELISA using recombinant rVP6 protein as the antigen. In brief, a 96-well ELISA plates were coated with 2 µg/mL of recombinant VP6 protein in carbonate buffer (pH 9.6) overnight at 4 °C (100 µL/well). Twofold dilutions of mouse sera (starting at a 1:100 dilution) or the lumen of the intestine (starting at a 1:20 dilution) were added to each well and incubated for 1 h at 37 °C. HRP-conjugated goat anti-mouse IgG (1:5000 dilution; Proteintech Group, Inc) and IgA (1:5000 dilution; Proteintech Group, Inc) were used to evaluate the the IgG and IgA antibody titers. The reaction was developed by 3,3′,5,5′-tetramethylbenzidine (TMB) solution, stopped with 2 N sulfuric acid, and absorbance was read at 450 nm. Positive reactions were defined as: the mean absorbance of reactions exceeded the absorbance of equal dilutions of the control sera by threefolds standard deviations.

### Acute toxicity analysis of the recombinant proteins and nanoparticles

Oral administration is one of the characteristics of ideal vaccine for its safety and low acute toxicity. Rotavirus and VP6 protein mainly induce mucosal immune response and has been widely studied in animal model without acute toxicity reported. Ferritin protein is widely found in organisms, including humans and mice, and the use of *Helicobacter pylori* ferritin protein has been proved to be free of autologous ferritin immunity in a previous study. Moreover, comparing to the intramuscularly vaccinated, oral administration used in our study is a safer way to avoid the toxic effect. Thus, we only evaluate the acute toxicity effects of VP6–ferritin nanoparticles on intestinal mucosa. Ten female BALB/c mice (3 weeks old; Experimental Animal Center, Guangxi Medical University, Nanning, China) were divided into two groups (5 in each group). One group was gavaged with 100 μL PBS containing 30 μg rVP6–ferritin proteins (this dose was the same as the immunogenicity assay) and the other group was gavaged with 100 μL PBS only. All of the mice were sacrificed on the second day (about 24 h later) after administration and the duodenums of the mice were separated and fixated by 4% PFA. Then the acute toxicity effects of VP6–ferritin nanoparticles on intestinal mucosa were analyzed by haematoxylin–eosin (HE) staining according to standard protocols [42].

### Construction of the gland expression vector

The rVP6–ferritin sequence was obtained from pUrVP6–ferritin using the specific primers rVP6–ferritin (Forward: GAATCGCGGATCCTCGAGATGAAGGTGCTGAT; Reverse: AGGGTCCGGATCCTCGAGTTAGCTCTTCCTGC). The rVP6–ferritin fragment was purified and inserted into the *Xho*I site of milk expression vector pBC1 using a Fast-Fusion™ kit (FFPC-C020, GeneCopoeia) according to the manufacturer’s instructions. The gland expression vector pBC_1_–rVP6–ferritin contains the goat β-casein promoter and signal peptide to drive transcription and secretion of the *rVP6*–*ferritin* gene.

### Generating and screening transgenic mice

The pBC_1_–rVP6–ferritin plasmid was purified with a PureLink™ HiPure Plasmid Filter Maxiprep Kit (K2100-17, Invitrogen) and digested with *Not*I and *Sal*I to remove the prokaryotic sequences. The target gene-containing fragment was purified with a QIA quick Gel Extraction Kit (Qiagen) according to the manufacturer’s instructions and diluted to 2 mg/mL with endotoxin-free TE buffer; it was then microinjected into the pronuclei of fertilized SPF FVB mouse strain zygotes according to standard protocols [43]. 5 founder transgenic mice (3 female and 2 male) were obtained and the founder female mice were mated with wild-type mice to expand the population. About half of the mice (6/11, 4/9, 7/13 respectively) born was transgenic in the F_1_ generation. Homozygous transgenic mice were obtained by inbreeding screening. A homozygous female transgenic mouse was mated with wild type mouse to generate the heterozygous mice, and these heterozygous mice were used in the following immunogenic responses analysis. Transgenic mice were screened by both PCR and Southern blot analysis with DNA samples obtained from tail biopsy. Primers specific to rVP6–ferritin were for PCR genotyping (Forward: CACTACCCTGCTGAATCTGG; Reverse: CCACGATGTTGTTGATGCTC). The Southern blotting used a DIG High Prime DNA Labeling and Detection Starter kit II (Roche) according to the manufacturer’s instructions. About 30 µg of mice genomic DNA from each sample was digested using the restriction enzymes *Bam*HI and *Nco*I. The prospective Southern blotting products were about 1.7 kb. A DNA sample from wild type mice was used as a negative control and the plasmid as digested by the restriction enzymes *Bam*HI and *Nco*I was used as a positive control.

### Expression analysis of VP6–ferritin in the milk via RT-PCR and western blotting

Three transgenic mice and wild type mice were sacrificed, and the tissues including mammal gland, heart, live, spleen, lung, kidney, and muscle were separated immediately. Total RNA of the tissues was isolated using TRizol reagent (Invitrogen), and was reverse transcribed into cDNA using a RevertAid First Strand cDNA Synthesis Kit (K1622, Thermo) according to the manufacturer’s instructions. Residual genomic DNA in total RNA was removed via DNase I treatment (Thermo) before the synthesis of cDNA. RT-PCR analysis used primers specific to *VP6*–*ferritin* (Forward: CACTACCCTGCTGAATCTGG; Reverse: CCACGATGTTGTTGATGCTC) or mice *β*-*actin* (Forward: GCACCACACCTTCTACAATGAGC; Reverse: TCGTTGCCAATAGTGATGACC), with the cDNA as the amplification templates.

Milk from transgenic mice and wild type mice was harvested on day 7 of lactation using an Automated Milker for Rat and Mouse (59150, Braintree Scientific, Inc). It was then diluted 1:10 with double distilled water, and defatted via centrifugation (4000×*g*, 20 min, 4 °C). The skim milk samples obtained were analyzed by western blot to confirm the expression of the recombinant protein.

### ELISA quantification of VP6–ferritin protein levels in transgenic mice

ELISA was used to determine the quantity of VP6–ferritin protein expressed in the milk. A 96-well ELISA plate was coated with mouse anti-VP6 antibody (Santa Crus: SC101363) in carbonate buffer (pH 9.6) overnight at 4 °C. The plate was washed three times with PBST and blocked with 3% BSA in PBST for 2 h at 37 °C. The skim milk samples were 1000-fold diluted before being added to the sample wells. Samples of known amounts of purified rVP6 protein from *E. coli* were used to generate a standard curve. Samples were added to the wells, and the plate was incubated for 1 h at 37 °C. After three washes, the plate was incubated with mouse anti-VP6 serum (1:1000, PA1-73015, Thermo) for 1 h at 37 °C, followed by additional washes and incubation with HRP-labeled goat anti-rabbit IgG (1:5000; Santa Cruz) for 1 h at 37 °C. The reaction was developed by 3,3′,5,5′-tetramethylbenzidine (TMB) solution, stopped with 2 N sulfuric acid, and absorbance was read at 450 nm. The expression level of the rVP6 antigen in the transgenic mice was quantified by comparison with the standard curve.

### Analysis of the immunogenic responses following breastfeeding from pBC1–rVP6–ferritin transgenic mice

The immunogenicity of rVP6–ferritin as delivered to pumps via breastfeeding from transgenic mice was analyzed by ELISA assays. The heterozygous female mice were mated with wild type male mice to ensure that both transgenic and non-transgenic pups can be breastfed by milk containing VP6–ferritin proteins. As control, the heterozygous male mice were mated with wild type female mice to ensure that both transgenic and non-transgenic pups can be breastfed by milk without VP6–ferritin proteins. Both transgenic and non-transgenic pups were breastfed for 3 weeks by rVP6–ferritin transgenic mothers or wild type mothers. On day 30 after weaning, ten mice in each group were sacrificed, and sera samples were collected and tested for IgG titers; small intestines were collected and homogenized in 0.01 M PBS for analysis of mucosal IgA. The levels of serum IgG and mucosal IgA antibodies by ELISA using recombinant rVP6 protein as the antigen, as described above.

### Challenge experiments

The transgenic mice were mated with wild type mice as above. Mouse pups were born after a 19 to 20-day gestation period. It is well-established that mice are only susceptible to rotavirus-induced diarrhea during the first 15 days of life [44], so 5-day-old pups breastfed by rVP6–ferritin transgenic mothers or wild type mothers were challenged with 200-fold TCID_50_ (half Tissue culture infectious dose) of simian rotavirus SA-11 (TCID_50_ = 10^5.4^). After SA-11 virus challenge, the anus of each pup was checked twice a day. The infected mice that did not exhibit watery feces during for either of the two daily checks were classified as non-diarrheal mice. The intensity of diarrhea was determined according to the color of anus and the amount of adhered feces. The proportion of pups showing diarrhea symptoms and the intensity of the diarrhea symptoms were observed for 10 days after challenge. The body weight of the pups was recorded using during the challenge experiment using an electronic scale.

### Statistical analysis

All of the data we obtained in this study were analyzed using one-way ANOVA followed by Duncan’s Multiple Comparison Tests or Student’s T-test as implemented in the SPSS 17 statistical software package.

## Additional file

**Additional file 1: Figure S1.** Nucleotide sequence of rVP6–ferritin used in this study. The sequences marked with red indicate the β-casein signal sequence; sequence in blue was the recombine VP6 gene and green indicated recombine ferritin gene. An S-G-G linker sequence was inserted between VP6 and ferritin sequence. **Figure S2.** Western blot analysis of recombinant protein expressed in *E. coli* with anti-his-tag antibodies. **Figure S3.** Molecular weight and hydrodynamic size analysis of the rVP6–ferritin subunit. A: Molecular weight of rVP6–ferritin subunit by mass spectroscopy; B: The size distribution determined by dynamic light scattering (DLS) and the hydrodynamic radius is showed in diagram. **Figure S4.** Acute toxicity effects of VP6–ferritin nanoparticles on intestinal mucosa by haematoxylin–eosin (HE) staining. **Figure S5.** Screen of transgenic mice and expression of rVP6–ferritin in different tissues of transgenic. A: PCR analysis of the transgenic mice; B: Southern blot analysis of the transgenic mice. C: Expression of rVP6–ferritin in transgenic mice analyzed by RT-PCR. NC: negative control; PC: positive control. The gland sample of wild type mice in lactating was used as negative control. **Figure S6.** Quantitation of rVP6–ferritin proteins expressed in transgenic mice milk by ELISA. Milk sample was diluted for 1000-folds before adding to the plate, and the concentration shown in the table is the original concentration of the recombine protein in the milk of transgenic mice. WT: milk sample from wild type mice; F1-2, F1-6, F1-16, F1-21, F1-28: milk sample from transgenic mice. The results are presented as mean ± SEM.

## References

[1] WHO (2013). Rotavirus vaccines WHO position paper: January 2013-Recommendations. Vaccine.

[2] Goveia MG, Rodriguez ZM, Dallas MJ, Itzler RF, Boslego JW, Heaton PM (2007). Safety and efficacy of the pentavalent human-bovine (WC3) reassortant rotavirus vaccine in healthy premature infants. Pediatr Infect Dis J..

[3] Ruiz-Palacios GM, Perez-Schael I, Velazquez FR, Abate H, Breuer T, Clemens SC (2006). Safety and efficacy of an attenuated vaccine against severe rotavirus gastroenteritis. N Engl J Med.

[4] Pliaka V, Kyriakopoulou Z, Markoulatos P (2012). Risks associated with the use of live-attenuated vaccine poliovirus strains and the strategies for control and eradication of paralytic poliomyelitis. Expert Rev Vaccines..

[5] Karch CP, Burkhard P (2016). Vaccine technologies: from whole organisms to rationally designed protein assemblies. Biochem Pharmacol.

[6] Greenberg HB, Estes MK (2009). Rotaviruses: from pathogenesis to vaccination. Gastroenterology.

[7] Esquivel FR, Lopez S, Guitierrez-x L, Arias C (2000). The internal rotavirus protein VP6 primes for an enhanced neutralizing antibody response. Arch Virol.

[8] Choi AH, McNeal MM, Basu M, Flint JA, Stone SC, Clements JD (2002). Intranasal or oral immunization of inbred and outbred mice with murine or human rotavirus VP6 proteins protects against viral shedding after challenge with murine rotaviruses. Vaccine..

[9] Burns JW, Siadat-Pajouh M, Krishnaney AA, Greenberg HB (1996). Protective effect of rotavirus VP6-specific IgA monoclonal antibodies that lack neutralizing activity. Science.

[10] Bremont M, Chabanne-Vautherot D, Vannier P, McCrae MA, Cohen J (1990). Sequence analysis of the gene (6) encoding the major capsid protein (VP6) of group C rotavirus: higher than expected homology to the corresponding protein from group A virus. Virology.

[11] Schwartz-Cornil I, Benureau Y, Greenberg H, Hendrickson BA, Cohen J (2002). Heterologous protection induced by the inner capsid proteins of rotavirus requires transcytosis of mucosal immunoglobulins. J Virol.

[12] Blazevic V, Lappalainen S, Nurminen K, Huhti L, Vesikari T (2011). Norovirus VLPs and rotavirus VP6 protein as combined vaccine for childhood gastroenteritis. Vaccine..

[13] Jalilvand S, Marashi SM, Shoja Z (2015). Rotavirus VP6 preparations as a non-replicating vaccine candidates. Vaccine..

[14] Moyle PM, Toth I (2013). Modern subunit vaccines: development, components, and research opportunities. ChemMedChem.

[15] Purcell AW, McCluskey J, Rossjohn J (2007). More than one reason to rethink the use of peptides in vaccine design. Nat Rev Drug Discov..

[16] Kanekiyo M, Wei CJ, Yassine HM, McTamney PM, Boyington JC, Whittle JR (2013). Self-assembling influenza nanoparticle vaccines elicit broadly neutralizing H1N1 antibodies. Nature.

[17] Li CQ, Soistman E, Carter DC (2006). Ferritin nanoparticle technology. A new platform for antigen presentation and vaccine development. Ind Biotechnol.

[18] Lonnerdal B (2003). Nutritional and physiologic significance of human milk proteins. Am J Clin Nutr.

[19] Lonnerdal B (2014). Infant formula and infant nutrition: bioactive proteins of human milk and implications for composition of infant formulas. Am J Clin Nutr.

[20] Wang J, Yang P, Tang B, Sun X, Zhang R, Guo C (2008). Expression and characterization of bioactive recombinant human alpha-lactalbumin in the milk of transgenic cloned cows. J Dairy Sci.

[21] van Berkel PH, Welling MM, Geerts M, van Veen HA, Ravensbergen B, Salaheddine M (2002). Large scale production of recombinant human lactoferrin in the milk of transgenic cows. Nat Biotechnol.

[22] Yang P, Wang J, Gong G, Sun X, Zhang R, Du Z (2008). Cattle mammary bioreactor generated by a novel procedure of transgenic cloning for large-scale production of functional human lactoferrin. PLoS ONE.

[23] Yang B, Wang J, Tang B, Liu Y, Guo C, Yang P (2011). Characterization of bioactive recombinant human lysozyme expressed in milk of cloned transgenic cattle. PLoS ONE.

[24] Rogier EW, Frantz AL, Bruno MEC, Wedlund L, Cohen DA, Stromberg AJ (2014). Secretory antibodies in breast milk promote long-term intestinal homeostasis by regulating the gut microbiota and host gene expression. Proc Natl Acad Sci.

[25] Andreas NJ, Kampmann B, Mehring Le-Doare K (2015). Human breast milk: a review on its composition and bioactivity. Early Hum Dev..

[26] Bosze Z, Hiripi L (2012). Recombinant protein expression in milk of livestock species. Methods Mol Biol.

[27] Houdebine L (2009). Production of pharmaceutical proteins by transgenic animals. Comp Immunol Microbiol Infect Dis.

[28] Soler E, Parez N, Passet B, Dubuquoy C, Riffault S, Pillot M (2007). Recombinant rotavirus inner core proteins produced in the milk of transgenic rabbits confer a high level of protection after intrarectal delivery. Vaccine..

[29] Patel M, Glass RI, Jiang B, Santosham M, Lopman B, Parashar U (2013). A systematic review of anti-rotavirus serum IgA antibody titer as a potential correlate of rotavirus vaccine efficacy. J Infect Dis.

[30] Cheuvart B, Neuzil KM, Steele AD, Cunliffe N, Madhi SA, Karkada N (2014). Association of serum anti-rotavirus immunoglobulin A antibody seropositivity and protection against severe rotavirus gastroenteritis: analysis of clinical trials of human rotavirus vaccine. Hum Vaccine Immunother..

[31] Feng N, Lawton JA, Gilbert J, Kuklin N, Vo P, Prasad BV (2002). Inhibition of rotavirus replication by a non-neutralizing, rotavirus VP6-specific IgA mAb. J Clin Invest..

[32] Corthsy B, Benureau Y, Perrier CM, Fourgeux C, Parez N, Greenberg H (2006). Rotavirus anti-VP6 secretory immunoglobulin A contributes to protection via intracellular neutralization but not via immune exclusion. J Virol.

[33] Bachmann MF, Zinkernagel RM (1997). Neutralizing antiviral B cell responses. Annu Rev Immunol.

[34] Wang L, Xing D, Le Van A, Jerse AE, Wang S (2017). Structure-based design of ferritin nanoparticle immunogens displaying antigenic loops of *Neisseria gonorrhoeae*. FEBS Open Bio..

[35] Soler E, Saux ASL, Guinut FDR, Passet B, Cohen R, Merle C (2005). Production of two vaccinating recombinant rotavirus proteins in the milk of transgenic rabbits. Transgenic Res.

[36] Feng H, Li X, Song W, Duan M, Chen H, Wang T (2017). Oral administration of a seed-based bivalent rotavirus vaccine containing VP6 and NSP4 induces specific immune responses in mice. Front Plant Sci..

[37] Dong JL, Zhou B, Sheng G, Wang T (2005). Transgenic tobacco expressing a modified VP6 gene protects mice against rotavirus infection. J Integr Plant Biol.

[38] Dong J, Liang B, Jin Y, Zhang W, Wang T (2005). Oral immunization with pBsVP6-transgenic alfalfa protects mice against rotavirus infection. Virology.

[39] Yu J, Langridge W (2003). Expression of rotavirus capsid protein VP6 in transgenic potato and its oral immunogenicity in mice. Transgenic Res.

[40] Truffi M, Fiandra L, Sorrentino L, Monieri M, Corsi F, Mazzucchelli S (2016). Ferritin nanocages: a biological platform for drug delivery, imaging and theranostics in cancer. Pharmacol Res.

[41] Arosio P, Ingrassia R, Cavadini P (2009). Ferritins: a family of molecules for iron storage, antioxidation and more. Biochim Biophys Acta.

[42] Fischer AH, Jacobson KA, Rose J, Zeller R (2008). Hematoxylin and Eosin staining of tissue and cell sections. Cold Spring Harb Protoc.

[43] Nagy A, Gertsenstein M, Vintersten K, Behringer R (2003). Manipulating the mouse embryo: a laboratory manual.

[44] Yu J, Langridge WH (2001). A plant-based multicomponent vaccine protects mice from enteric diseases. Nat Biotechnol.

