# Supplementary material for: A milk-based self-assemble rotavirus VP6–ferritin nanoparticle vaccine elicited protection against the viral infection
Source: J Nanobiotechnology. 2019 Jan 22;17:13. doi: 10.1186/s12951-019-0446-6 (PMC6341625; doi:10.1186/s12951-019-0446-6)
Supplement: Supplementary file 1 — Additional file 1: Figure S1. Nucleotide sequence of rVP6–ferritin used in this study. The sequences marked with red indicate the β-casein signal sequence; sequence in blue was the recombine VP6 gene and green indicated recombine ferritin gene. An S-G-G linker sequence was inserted between VP6 and ferritin sequence. Figure S2. Western blot analysis of recombinant protein expressed in E. coli with anti-his-tag antibodies. Figure S3. Molecular weight and hydrodynamic size analysis of the rVP6–ferritin subunit. A: Molecular weight of rVP6–ferritin subunit by mass spectroscopy; B: The size distribution determined by dynamic light scattering (DLS) and the hydrodynamic radius is showed in diagram. Figure S4. Acute toxicity effects of VP6–ferritin nanoparticles on intestinal mucosa by haematoxylin–eosin (HE) staining. Figure S5. Screen of transgenic mice and expression of rVP6–ferritin in different tissues of transgenic. A: PCR analysis of the transgenic mice; B: Southern blot analysis of the transgenic mice. C: Expression of rVP6–ferritin in transgenic mice analyzed by RT-PCR. NC: negative control; PC: positive control. The gland sample of wild type mice in lactating was used as negative control. Figure S6. Quantitation of rVP6–ferritin proteins expressed in transgenic mice milk by ELISA. Milk sample was diluted for 1000-folds before adding to the plate, and the concentration shown in the table is the original concentration of the recombine protein in the milk of transgenic mice. WT: milk sample from wild type mice; F1-2, F1-6, F1-16, F1-21, F1-28: milk sample from transgenic mice. The results are presented as mean ± SEM. [file 12951_2019_446_MOESM1_ESM.docx]

Additional figures for

“A mammal gland-based self-assembling rotavirus nanoparticle vaccine induced protection against the viral infection in mice”

Zhipeng Li ^a,^ *, Kuiqing Cui ^a,^ *, Hong Wang ^b,^ *, Fuhang Liu ^a^, Kongwei Huang ^a^, Zhaojun Duan ^b^, Fengchao Wang ^c^, Deshun Shi ^a, †^, Qingyou Liu ^a, †^

^a^ State Key Laboratory for Conservation and Utilization of Subtropical Agro-Bioresources, Guangxi University, 530004, Nanning, Guangxi, China;

^b^ National Institute for Viral Disease Control and Prevention, China CDC, 102206, Beijing, China;

^c^ National Institute of Biological Sciences (NIBS), 102206, Beijing, China.

*These authors contributed equally to this work.

† Correspondence should be addressed to Deshun Shi (ardsshi@gxu.edu.cn) or Qingyou Liu ([qyliu-gene@qq.com](mailto:qyliu-gene@qq.com)).

**Figures**


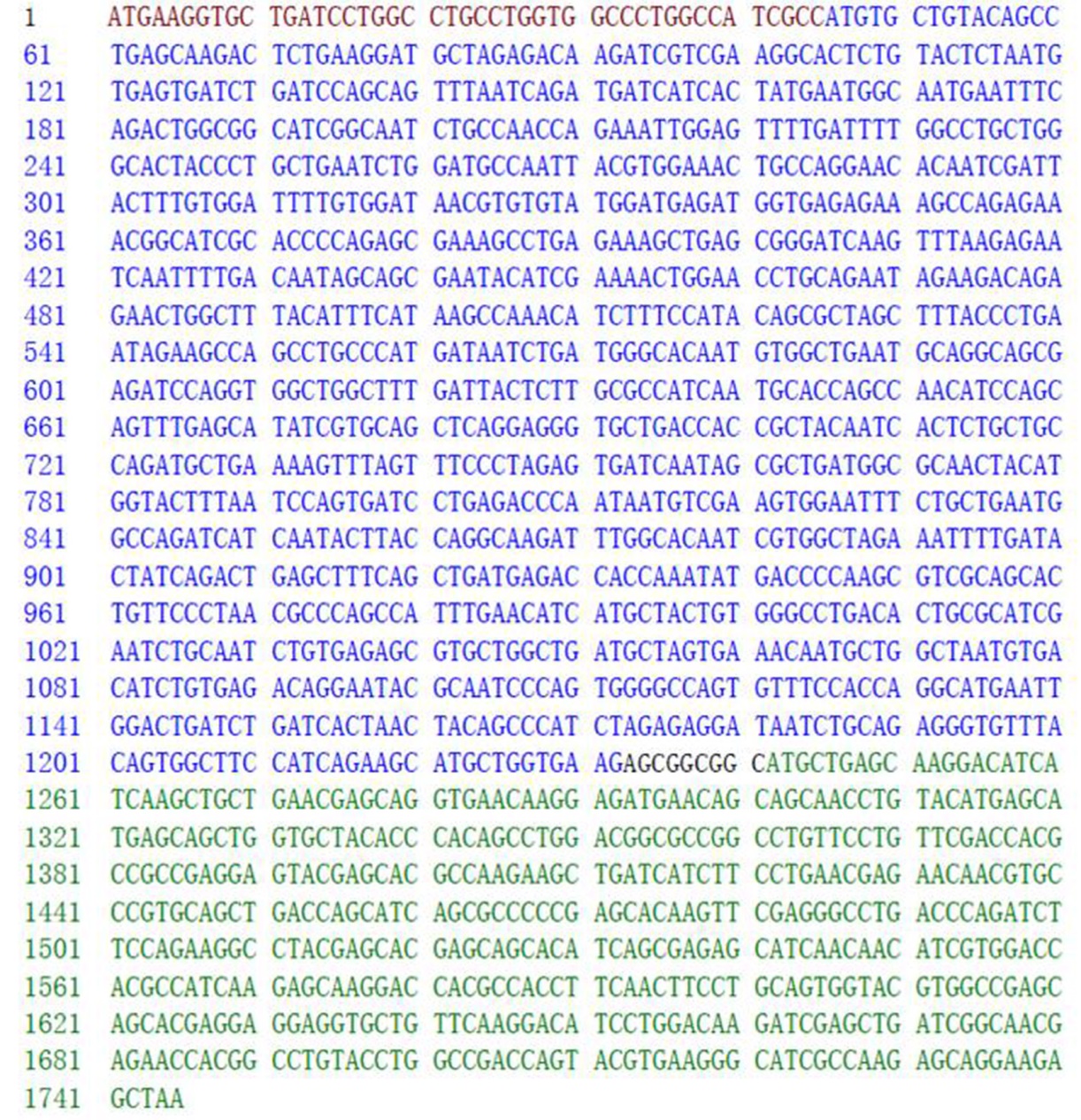


**Fig.S1** **Nucleotide sequence of rVP6-ferritin used in this study.** The sequences marked with red indicate the β-casein signal sequence; sequence in blue was the recombine *VP6* gene and green indicated recombine *ferritin* gene. An S-G-G linker sequence was inserted between VP6 and ferritin sequence.


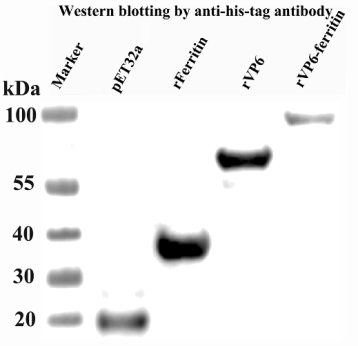


**Fig.S2** **Western blot analysis of recombinant protein expressed in E. coli** **with anti-his-tag antibodies.**


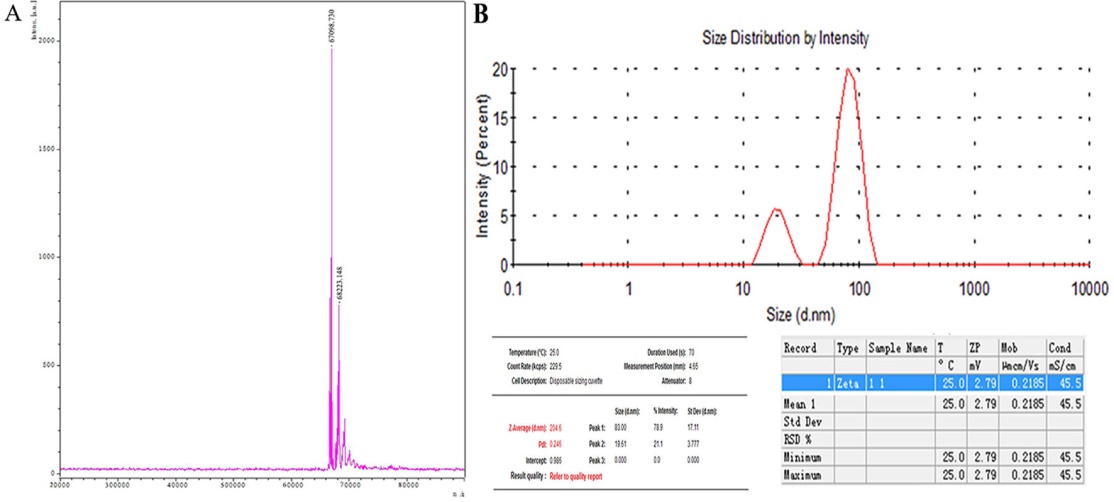


**Fig.S3 Molecular weight and hydrodynamic size analysis of the rVP6-ferritin subunit.** A: Molecular weight of rVP6-ferritin subunit by mass spectroscopy; B: The size distribution determined by dynamic light scattering (DLS) and the hydrodynamic radius is showed in diagram.


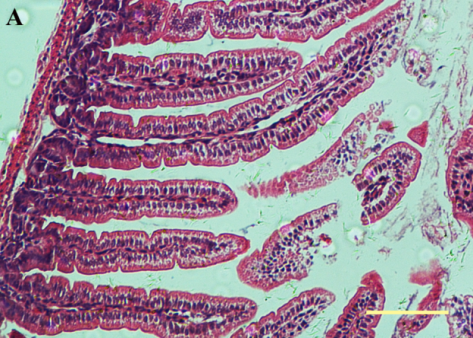

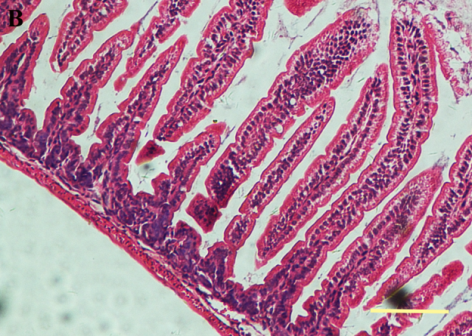


**Fig.S4 Acute toxicity effects of VP6-ferritin nanoparticles on intestinal mucosa by haematoxylin-eosin (HE) staining.** A: Intestinal of mouse gavaged by 100 μL PBS containing 20 μg of rVP6-ferritin nanoparticles; B: Intestinal of mouse gavaged by 100 μL PBS only. Scale bar indicated 100 μm.


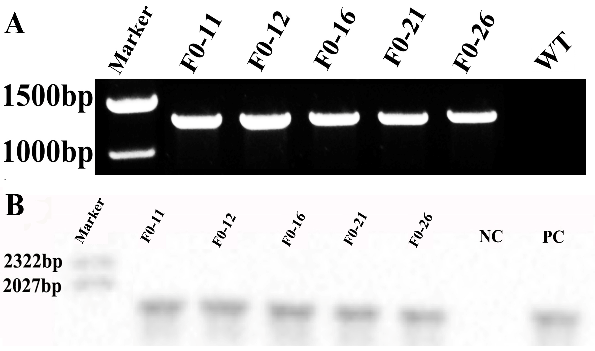




**Fig.S5** **Screen of transgenic mice and expression of rVP6-ferritin in different tissues of transgenic.** A: PCR analysis of the transgenic mice; B: Southern blot analysis of the transgenic mice. C: Expression of rVP6-ferritin in transgenic mice analyzed by RT-PCR. NC: negative control; PC: positive control. The gland sample of wild type mice in lactating was used as negative control.


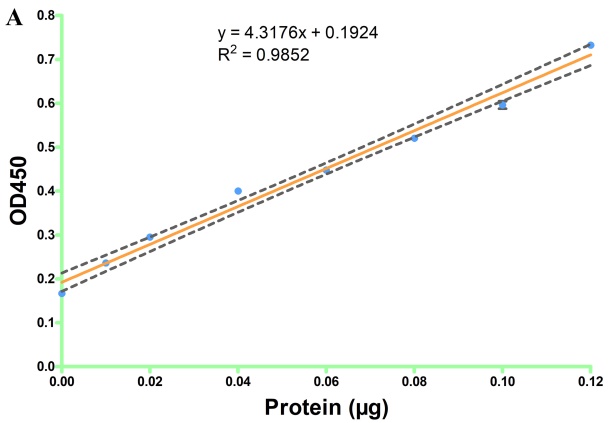

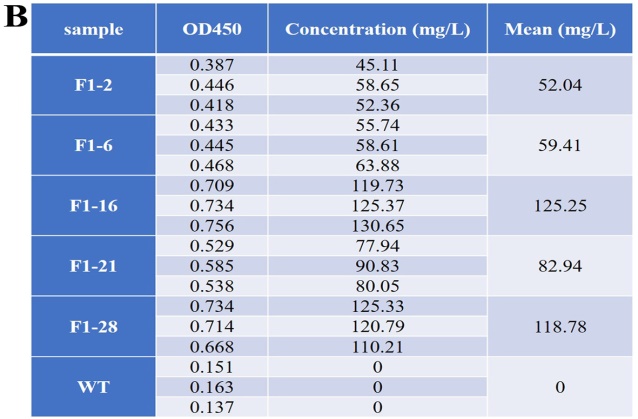


**Fig.S6** **Quantitation of rVP6-ferritin proteins expressed in transgenic mice milk by ELISA.** Milk sample was diluted for 1000 folds before adding to the plate, and the concentration shown in the table is the original concentration of the recombine protein in the milk of transgenic mice. WT: milk sample from wild type mice; F1-2, F1-6, F1-16, F1-21, F1-28: milk sample from transgenic mice. The results are presented as mean ± SEM.
